# Estimating Blood Volume Loss With Wireless, Wearable Ultrasound: A Pilot Investigation

**DOI:** 10.1093/milmed/usag109

**Published:** 2026-08-06

**Authors:** Jon-Emile S Kenny, Melissa Hebscher, Tracy Savery, Sarah Atwi, Daniel Lachner-Piza, Andrew M Eibl, Joseph K Eibl, Soumya Vungarala, Niki M Dietz, Chul-Ho Kim, Bruce D Johnson

**Affiliations:** Department of Clinical Research, Health Sciences North Research Institute Walford Site, Greater Sudbury, ON P3E 2H9, Canada; Department of Engineering, Flosonics Medical, Toronto, ON M5V 2Y1, Canada; Department of Engineering, Flosonics Medical, Greater Sudbury, ON P3E 2H9, Canada; Department of Engineering, Flosonics Medical, Toronto, ON M5V 2Y1, Canada; Department of Engineering, Flosonics Medical, Toronto, ON M5V 2Y1, Canada; Department of Clinical Research, Health Sciences North Research Institute Walford Site, Greater Sudbury, ON P3E 2H9, Canada; Department of Clinical Research, Health Sciences North Research Institute Walford Site, Greater Sudbury, ON P3E 2H9, Canada; Department of Cardiovascular Medicine, Mayo Clinic, Rochester, MN 55905, United States; Anesthesiology and Perioperative Medicine, Mayo Clinic, Rochester, MN 55905, United States; Human Integrative and Environmental Physiology Laboratory, Mayo Clinic, Rochester, MN 55905, United States; Human Integrative and Environmental Physiology Laboratory, Mayo Clinic, Rochester, MN 55905, United States

## Abstract

**Introduction:**

Detecting and estimating blood volume loss are important for the diagnosis and management of life-threatening hemorrhage. There is a direct relationship between percent blood volume loss (BVL_%_) and falling in stroke volume (SV); furthermore, there is a direct relationship between diminishing SV and the corrected flow time of the carotid artery (ccFT). Therefore, we hypothesized that change in ccFT (ccFT_Δ_) would directly relate with BVL_%_ in a phlebotomy paradigm.

**Materials and Methods:**

A prospective, convenience sample of healthy volunteers was studied. Volunteers underwent blood donation protocol in a physiology lab. A wireless, wearable Doppler ultrasound system monitored ccFT during blood donation. Traditional vital signs were also measured throughout. The relationship between traditional vital signs as well as ccFT_Δ_ were compared to BVL_%_ using 2 mixed linear regression models.

**Results:**

Data from 20 subjects and blood donations comprising 46,123 cardiac cycles are included. On average 413.5 ± 39 mL of blood were drawn representing 8.6 ± 1.6% BVL_%_. There was a significant and strong linear relationship between BVL_%_ and ccFT_Δ_ during blood draw with the regression slope demonstrating that for every 1 ms reduction in ccFT there was a 0.4% reduction in blood volume.

**Conclusions:**

In healthy subjects and when blood loss is the sole mechanism for falling SV, we estimate that for every 1 ms reduction in ccFT, there is an approximate 0.4 BVL_%_. Validation of these results in patients with traumatic hemorrhage is underway.

## INTRODUCTION

Detecting traumatic blood loss is traditionally accomplished via clinical history and vital signs,^1^ though point of careultrasound imaging is increasingly used.^2^ Yet, without ultrasound image supplementation, vital signs alone are generally insensitive for diagnosing cryptic hemorrhage and only weakly relate with percent blood volume loss (BVL_%_).^1^ Estimating BVL_%_ is clinically challenging with numerous research techniques available but few easily applied to patients with acute traumatic injuries.^3^ Therefore, an objective assessment of BVL_%_, especially monitored over time, could help triage and treat patients with life-threatening hemorrhage.

We have previously described a novel, wireless, and wearable continuous wave Doppler ultrasound that continuously measures surrogates of SV in the common carotid artery.^4-8^ Using this new ultrasound technology, we have shown that a 10% fall in stroke volume (SV) correlates with a 5- to 10-ms reduction in the corrected flow time of the common carotid artery (ccFT).^9-11^ Furthermore, it has been shown that BVL_%_ can be estimated as approximately one-half the percent fall in SV during models of central hypovolemia and exsanguination.^12^ Given these previously reported values, we hypothesized that for every 0.25 to 0.5% reduction in blood volume (i.e., 0.25-0.5 BVL_%_), ccFT would fall, on average, by 1 ms. To test this hypothesis, we measured the change in ccFT (ccFT_Δ_) and BVL_%_ during phlebotomy of healthy volunteers.

## MATERIALS AND METHODS

### Clinical Setting

The Research Ethics Board of the Mayo Clinic approved this study. A convenience sample of adult patients recruited by a physiology laboratory comprise this analysis. Exclusion criteria were: blood donation within 112 days, hemoglobin <13.0 g/dL for men and <12.0 g/dL for women, under the age of 18 or over 50, systolic blood pressure <90 mm Hg or >180 mm Hg, diastolic blood pressure >100 mm Hg, current smokers, BMI >38 kg/m^2^, known or planned pregnancy.

### Blood Donation and Blood Volume Estimation

Subjects remained in the sitting position in a blood donation chair. Subjects underwent blood draw procedure. Blood was removed slowly into a blood bag over 8 to 12 minutes (approximately 50 mL/min) via a 16- or 18-gauge intravenous catheter. The blood volume withdrawn was not to exceed 13% of whole blood volume with a maximum withdrawal of 450 mL ±10%. The subject’s whole blood volume was estimated by the equation of Nadler et al.^13^

### Physiological Monitoring

**Figure 1.**
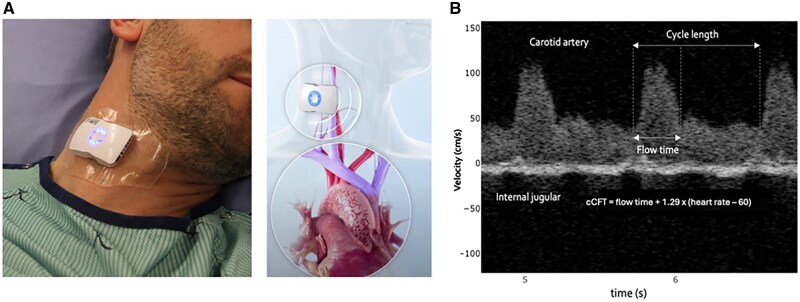
(A) The wireless Doppler ultrasound in place on a patient’s neck. The device is placed over the common carotid artery and internal jugular vein and communicates wirelessly with a nearby tablet. (B) Example of carotid Doppler spectrogram as it appears in the graphical user interface. Features from the device used to calculate ccFT using Wodey’s equation and labeled in white.

A wearable Doppler ultrasound system (Flosonics Medical, Sudbury, Canada) was placed on the neck over the common carotid artery.^9^ In all subjects the common carotid Doppler pulse was detected by placing the device adjacent to the thyroid cartilage and moved laterally until a displayed spectrogram and audible Doppler shift consistent with the common carotid artery was obtained; then the wearable device was adhered in place using its incorporated adhesive. Data from the device is transmitted via Bluetooth to an iOS graphical interface. The ccFT was determined by Wodey’s equation as:


ccFT=Flow Time+1.29 x (Heart Rate-60)

where flow time is the duration of systole in seconds and heart rate is 60/cycle length (Figure 1B).

In addition to the wearable ultrasound, the Nexfin, finger-cuff, non-invasive hemodynamic monitor (Edwards Lifesciences, Irvine, CA, United States) was worn to measure continuous vital signs throughout the entire blood draw. Baseline values were recorded over a 2-minute period before beginning blood draw. Continuous monitoring persisted through the blood draw and for an additional 5 minutes, post-procedure.

### Data Alignment and Preprocessing

Carotid Doppler and Nexfin hemodynamic data were temporally aligned to ensure accurate synchronization for analysis. Alignment was performed by calculating the time shift with the greatest correlation between carotid Doppler and Nexfin heart rate for each subject. Doppler and Nexfin data were then examined for outliers in ccFT, heart rate, Nexfin stroke volume (SV), and Nexfin mean arterial pressure (MAP). Outliers were identified and excluded based on being more than 2 standard deviations from the subject’s own mean, for each variable of interest. Our analysis reflects the underlying trend during a gradual 8 to 12 minute blood draw rather than single cardiac cycle fluctuations. To mitigate beat-to-beat variability, respiratory variability, and potential measurement noise, a rolling mean smoothing filter of 30 cardiac cycles was then applied to the cleaned data to reduce cycle-to-cycle variability.

Percent blood volume loss (BVL_%_) was estimated by dividing the total volume of blood withdrawn for each subject by their estimated total blood volume. Total blood volume was estimated based on sex, height, and weight using Nadler et al’s formula^13^ (Men: Blood Volume = [0.3669 × Height^3^] + [0.03219 × Weight] + 0.6041; Women: Blood Volume = [0.3561 × Height^3^] + [0.03308 × Weight] + 0.1833). Within the blood draw period, cumulative BVL_%_ over time was calculated by distributing the total BVL_%_ proportionally across the duration of the blood draw, assuming a constant rate of withdrawal. Change in ccFT (ccFT_Δ_) was computed as the difference between the baseline average and each time point during blood draw. Change in MAP (MAP_Δ_) and stroke volume (SV_Δ_) from Nexfin were calculated in the same way, as the difference between the baseline average and the value at each time point during blood draw.

### Statistical Analysis

Change in traditional vital signs as well as ccFT_Δ_, were compared to BVL_%_ using mixed-effects linear regression models. The first model assessed whether ccFT_Δ_ predicted BVL_%_ at the subject level, with BVL_%_ as the dependent variable, ccFT_Δ_ as the fixed effect predictor, and a random intercept for subject ID. The second model assessed whether MAP_Δ_ from Nexfin predicted BVL_%_ at the subject level, with BVL_%_ as the dependent variable, MAP_Δ_ as the fixed effect predictor, and a random intercept for subject ID. Individual regression models were also run for each subject and median regression coefficients and slopes were reported to quantify associations. Additionally, absolute values of all variables at baseline and the final 60 seconds of the blood draw were compared using paired, 2-tailed Student’s *t*-tests; 95th confidence intervals were reported for linear regressions.

To control for the effect of confounders across Doppler- and Nexfin-based metrics, we ran a series of mixed-effects regressions. First, all Doppler measurements between blood-draw timepoints and baseline (i.e., ccFT_Δ_, Shock-Index_Δ_ and Heart-Rate_Δ_) were used as independent predictors of BVL%. The performance from each of these separate regression models was compared using the Akaike Information Criterion (AIC). Second, Doppler measurements were combined in a stepwise manner and used in a series of multivariate predictors of the BVL%; subsequent change in performance was quantified using the AIC. The same stepwise analysis and performance assessment was run on Nexfin measurements (i.e., MAP_Δ_, SV_Δ_, HR_Δ_, SpO_2Δ_).

## RESULTS

We enrolled 21 subjects; 1 subject was excluded for low hemoglobin levels; data from 20 subjects and blood donations comprising 46,123 cardiac cycles are included. For analyses with Nexfin measures (MAP, SV), 1 subject was excluded because of Nexfin signal dropout during blood draw. Table 1 shows the baseline subject characteristics before blood draw and during the final 60 seconds of blood draw, with *P* values for the comparison between baseline and the final 60 seconds of blood draw. On average 413.5 ± 39 mL of blood were drawn, representing 8.6 ± 1.6% blood volume.

**Table 1.** Subject Characteristics and Hemodynamic Change During Phlebotomy (*n* = 20)

Baseline value ± standard deviation			
Age (yr)	31.9 ± 6.1		
Sex (female)	9(11)		
Weight (kg)	80.8 ± 17.0		
Height (cm)	173.9 ± 9.4		
BMI (kg/m^2^)	26.6 ± 4.3		
Estimated blood volume (L)	4.9 ± 0.9		
Systolic blood pressure (mm Hg)^a^	122.4 ± 10.0	Final minute of blood draw	
Diastolic blood pressure (mm Hg)^a^	78.1 ± 7.9	Mean ± SD	*P* value, baseline vs. blood draw
Mean arterial pressure (mm Hg)^b^	94.2 ± 12.0	92.6 ± 12.9	.33
Oxygen saturation (%)^b^	97.6 ± 1.3	97.2 ± 1.6	.13
Heart rate (bpm)^b^	69.7 ± 11.5	74.8 ± 12.0	<.01
Corrected flow time (ms)^b^	310.6 ± 13.5	300.9 ± 14.2	<.01
Doppler Shock Index^b^,^c^	0.22 ± 0.04	0.25 ± 0.05	<.01
Stroke volume (mL)^b^	109.3 ± 19.9	103.7 ± 19.2	<.01

a Single measurement taken at start of baseline.

b Continuous measurement throughout the baseline phase, averaged.

c Heart rate divided by corrected flow time in milliseconds.

**Figure 2.**
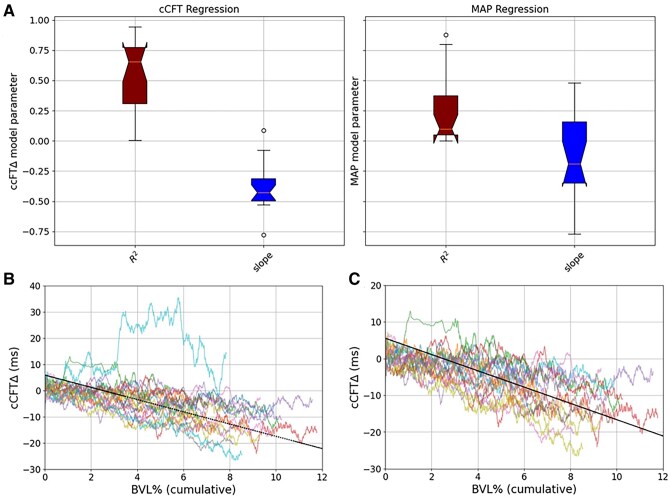
(A) (Left) the relationship between ccFT_Δ_ vs. BVL_%_ (median *R*^2^ = 0.65, slope = −0.43) and (right) MAP vs. BVL_%_ (median *R*^2^ = 0.09, slope = −0.19) from individual linear regressions. (B) Regression of ccFT_Δ_ vs. BVL_%_ is plotted. Colors represent individual subjects. Dotted line represents the median linear regression line. (C) Regression of ccFT_Δ_ vs. BVL_%_ excluding 1 outlier subject with large increase of ccFT_Δ_ in response to BVL from the ccFT_Δ_ vs. BVL_%_ analysis (median *R*^2^ = 0.70, slope = −0.45).

A mixed linear regression model showed a significant negative association between ccFT_Δ_ and cumulative blood volume loss (β = −0.29 and CI= [−0.30 to −0.29], standard error (SE) <0.01, *z* = −102.8, *P* < .001), where the model fit the data well (log-likelihood = −41,334) (Figure 2A). As seen in Figure 2B, there was 1 subject who showed a large increase in ccFT_Δ_ in response to the blood draw phase. Excluding that subject from the mixed linear regression model resulted in a stronger negative association (β = −.40 and CI = [−0.40 to −0.39], SE < 0.01, *z* = −144.80, *P* < .001) (Figure 2C). For every 1 ms decrease in ccFT, there was an estimated 0.4 BVL_%_. Within-subject regressions to quantify associations similarly showed an approximate 0.4% blood volume loss for each 1 ms drop in ccFT (median *R*^2^ = 0.65 and CI = [0.35 to 0.74], median slope = −0.43 and CI = [−0.49 to −0.33]), with a stronger association after excluding the 1 outlier subject (median *R*^2^ = 0.70 and CI = [0.39 to 0.76], slope = −0.45 and CI = [−0.50 to −0.35]).

Nexfin MAP_Δ_ was significantly associated with blood volume loss percentage, but to a lesser degree than the association with ccFT_Δ_ (β = −.15, CI = [−0.16 to −0.14], SE <0.01, *z* = −27.40, *P* < .001; log-likelihood = −43,767). Within-subject regressions with MAP to quantify associations showed lower median *R*^2^ and slope compared to the association with ccFT_Δ_ (median *R*^2^ = 0.09 and CI = [0.05 to 0.37], slope = −0.19 and CI = [−0.36 to 0.09]; Figure 2A).

Paired *t*-tests comparing average baseline measures to the final minute of blood draw revealed significant differences in heart rate (*t* = −5.02, *P* < .001), ccFT (*t* = 6.04, *P < .*001), Doppler Shock Index (*t* = −6.92, *P < .*001), and stroke volume (*t* = 3.13, *P < .*01). No differences were observed from baseline to the final minute of blood draw for MAP (*t* = 1.00, *P = .*33) or oxygen saturation (*t* = 1.60, *P = .*13) (Table 1).

When controlling for confounders, ccFT_Δ_ as a single fixed-effect provided the best prediction of BVL_%_ (*R*^2^ = 0.54, AIC = 74,108, *P < .*001, *F*-statistic = 17,665). Following a stepwise regression, a combination of ccFT_Δ,_ Shock-Index_Δ_, and Heart-Rate_Δ_ showed the best overall performance (*R*^2^ = 0.67, AIC = 68,032, *P < .*001, *F*-statistic = 10,627). Analogous Nexfin stepwise regressions results showed the combination of MAP_Δ_, SV_Δ_, HR_Δ_, SpO_2Δ_ (*R*^2^ = 0.64, AIC = 72,854, *P < .*001, *F*-statistic = 5,058).

## DISCUSSION

We observed a significant and strong linear relationship between BVL_%_ and ccFT_Δ_ during blood removal. Our findings are notable because they are consistent with previous investigations and provide evidence that following ccFT_Δ_ during hemorrhage could help gauge BVL_%_ as a triage and therapeutic tool for patients during active hemorrhage. Furthermore, we note that traditional vital signs like mean arterial pressure correlate less strongly with BVL_%_, suggesting that directly assessing blood flow surrogates from Doppler ultrasound are more sensitive for detecting cryptic blood loss.

Mackenzie et al^14^ were the first to describe ccFT_Δ_ obtained via traditional, hand-held Doppler ultrasound during blood donation; our observations with wireless, wearable Doppler ultrasound are nearly identical. In their investigation, 70 subjects donated, on average, roughly 450 mL of blood and ccFT fell by about 20 ms. If 450 mL represents approximately 10% blood volume loss, their observations imply that for every 0.5 BVL_%_ there is a 1 ms reduction in ccFT. Fundamentally, the mechanism here is that diminished preload reduces stroke volume which shortens left ventricular ejection time.^15^ In line with this reasoning, other investigators have found a strong, direct relationship between volume removed during hemodialysis and falling ccFT.^16^^,^^17^ Nevertheless, volume loss during hemodialysis is not completely interchangeable with the physiology of whole blood loss.

The rationale behind measuring SV (or a surrogate like the ccFT) in traumatically injured patients is that falling blood pressure is a lagging indicator of hemodynamic deterioration; blood pressure is buoyed by a compensatory increase in systemic vascular resistance secondary to adrenergic tone. Consistent with this reasoning, previous investigations^18-24^ have shown that non-surviving trauma patients who present to the emergency department (ED) had significantly reduced SV as compared to those who survived. Accordingly, early assessment of SV surrogates like the ccFT might raise clinical concern earlier in care and expedite definitive therapy.

Finally, we demonstrate that traditional vital signs such as mean arterial pressure change (MAP_Δ_) showed a much weaker relationship with BVL_%_. This is not a new finding but reinforces the known limitation of using blood pressure to infer fall in SV in the face of adrenergic reflexes. Other investigators have integrated basic vital signs into summary scores, for instance, the compensatory reserve index (CRI), which are much better at predicting hemodynamic deterioration,^25^ however we did not compare carotid Doppler ultrasound to the CRI.

This investigation has a number of limitations. First, we studied only healthy volunteers. Extrapolation of this data to other patient populations such as the elderly or those with significant cardiopulmonary co-morbidities should be done with caution. Indeed, aging may affect these findings as vascular compliance and ventricular-arterial coupling may shift. Cardiovascular co-morbidities, such as heart failure, arrhythmias (e.g., atrial fibrillation), hypertension, and valvular diseases all may alter Doppler waveform morphology and impact ccFT; these influences on ccFT are described in a recent review.^15^ Similarly, baseline volume status, sympathetic tone, respiratory patterns,^26^ and device placement variability may influence absolute ccFT. Although we mitigate certain factors by using relative metrics (baseline vs. blood draw timepoints) rather than absolutes, and averaging dozens of consecutive cardiac cycles, further stratification of response by cohort is necessary. Furthermore, the sensitivity of traditional vital signs at detecting BVL_%_ is probably poorest in healthy volunteers because of their robust compensatory response. That we found a clear signal in those who maintained their blood pressure during blood loss is noteworthy. Second, it was a small sample size. However, this was a feasibility study, and our results are in-line with that of Mackenzie et al^14^ described above. Third, ccFT_Δ_ as an indicatory of BVL_%_ holds only when falling preload reduces SV. Although this is highly likely in experimental blood removal, in hemorrhage models such as lower body negative pressure or when patients are being evaluated for a mechanism of injury at high risk of significant blood loss, SV can fall in the clinical arena for multiple reasons (e.g., intrinsic cardiac dysfunction, obstructive shock, etc.). Therefore, diminishing ccFT cannot be universally thought of as an indication of blood volume loss. Conversely, at least 1 outlier had a significant increase in SV and ccFT during BVL, which probably represents a robust compensatory recruitment of venous blood, for instance, from the splanchnic circulation during donation; therefore, it is unlikely that the slope relating ccFT_Δ_ to BVL is universal. Fourth, ccFT_Δ_ as a marker of changing SV holds only when afterload and cardiac contractility are relatively constant^15^ (i.e., falling preload is the primary driver diminishing SV). To the extent that downstream vasoconstriction raises afterload, decreasing ccFT in this model may be blunted relative to true SV loss. However, in healthy volunteer models of mild-to-severe central blood volume loss and in the supine position, we found a linear trend between ccFT and SV.^27^^,^^28^ Furthermore, in a small, pilot study, supine trauma patients who required massive transfusion activations had very low absolute ccFT (i.e., <270 ms) measured by the wearable Doppler system.^29^ Finally, because of the nature of this study, we do not know the effect of commonly used sedatives or anesthetics on the slope relating ccFT_Δ_ to BVL_%_. We, and others, have shown that ccFT_Δ_ does reliably track SV change in sedated and/or anesthetized patients,^8^^,^^30^^,^^31^ however, the relationship between SV change and BVL_%_ might be different in anesthetized patients.

In conclusion, the wearable Doppler ultrasound obtained continuous ccFT measurements during an 8- to 12-minute blood volume removal protocol culminating in roughly 8% blood volume loss across all subjects. The regression slope between BVL_%_ and ccFT_Δ_ suggests that when blood loss is the sole mechanism for falling stroke volume, for every 0.4% blood volume loss, there is a 1 ms reduction in ccFT. Further validation in patients with hemorrhage is planned.

## Data Availability

Available upon request.

## INSTITUTIONAL CLEARANCE

Institutional approval granted by Institutional Ethics Committee of the Mayo Clinic, above.
